# ‘We did everything we could’– a qualitative study exploring the acceptability of maternal-fetal surgery for spina bifida to parents

**DOI:** 10.1002/pd.5996

**Published:** 2021-07-18

**Authors:** Neeltje Crombag, Adalina Sacco, Bernadette Stocks, Philippe De Vloo, Johannes van der Merwe, Katie Gallagher, Anna David, Neil Marlow, Jan Deprest

**Affiliations:** 1 Department of Development and Regeneration Cluster Woman and Child, Biomedical Sciences, KU Leuven, Leuven, Belgium; 2 Elizabeth Garrett Anderson Institute of Women’s Health, University College London, London, UK; 3 NHS England and NHS Improvement, London, UK; 4 Department of Neurosurgery, University Hospitals Leuven, Leuven, Belgium; 5 Department of Obstetrics and Gynecology, Division Woman and Child, University Hospitals Leuven, Leuven, Belgium; 6 National Institutes for Health, University College London Hospitals Biomedical Research Centre, London, UK

## Abstract

**Objective:**

To explore the concepts and strategies parents employ when considering maternal-fetal surgery (MFS) as an option for the management of spina bifida (SB) in their fetus, and how this determines the acceptability of the intervention.

**Methods:**

A two-centre interview study enrolling parents whose fetuses with SB were eligible for MFS. To assess differences in acceptability, parents opting for MFS (*n* = 24) were interviewed at three different moments in time: prior to the intervention, directly after the intervention and 3-6 months after birth. Parents opting for termination of pregnancy (*n* = 5) were interviewed only once. Themes were identified and organised in line with the framework of acceptability.

**Results:**

To parents opting for MFS, the intervention was perceived as an opportunity that needed to be taken. Feelings of parental responsibility drove them to do anything in their power to improve their future child’s situation. Expectations seemed to be realistic yet were driven by hope for the best outcome. None expressed decisional regret at any stage, despite substantial impact and, at times, disappointing outcomes. For the small group of participants, who decided to opt for termination of pregnancy (TOP), MFS was not perceived as an intervention that substantially could improve the quality of their future child’s life.

**Conclusion:**

Prospective parents opting for MFS were driven by their feelings of parental responsibility. They recognise the fetus as their future child and value information and care focusing on optimising the child’s future health. In the small group of parents opting for TOP, MFS was felt to offer insufficient certainty of substantial improvement in quality of life and the perceived severe impact of SB drove their decision to end the pregnancy.

## 1 Introduction

Maternal-fetal surgery (MFS) for open spina bifida (SB) has the potential to improve long-term outcomes but at significant procedure-related fetal and maternal risk.^1–5^ Following antenatal diagnosis of open SB, prospective parents face a range of uncertainties regarding the future of their unborn child, and the options provided pose major ethical challenges.^6^ In this situation parents frequently choose termination of pregnancy (TOP), as opposed to expectant prenatal management (EM) followed by postnatal assessment and surgery.^7,8^ MFS provides now an additional third option in which antenatal coverage of the fetal neural tissue is undertaken to prevent further damage. This intervention improves medium-term neurological outcomes, but risks premature membrane rupture, preterm delivery and rarely fetal death. For the mother, the procedure can cause perioperative morbidity and complications and risks uterine dehiscence in the current or future pregnancies, for which an elective caesarean section is required.^1–3,9,10^ For parents, balancing these competing risks is challenging.

Successful implementation and evaluation of an intervention not only requires it to be effective, but also for it to be acceptable to those undergoing the intervention.^11^ Acceptability can be defined as a construct that reflects the extent to which people receiving a healthcare intervention consider it appropriate, based on anticipated or experienced responses to the intervention.^12^ Several aspects of the management pathways of fetal SB may be perceived to affect the acceptability of the intervention to parents. Little is known about the processes that parents experience in choosing a specific course, particularly as MFS is an option that could improve the long-term outcome but which also carries a significant risk to the pregnant woman and her fetus. This study investigates the concepts and strategies parents employ when considering MFS as an option for the management of SB in their fetus, and how this determines the acceptability of the intervention.

## 2 Methods

### 2.1 Theoretical Framework

**Figure 1:**
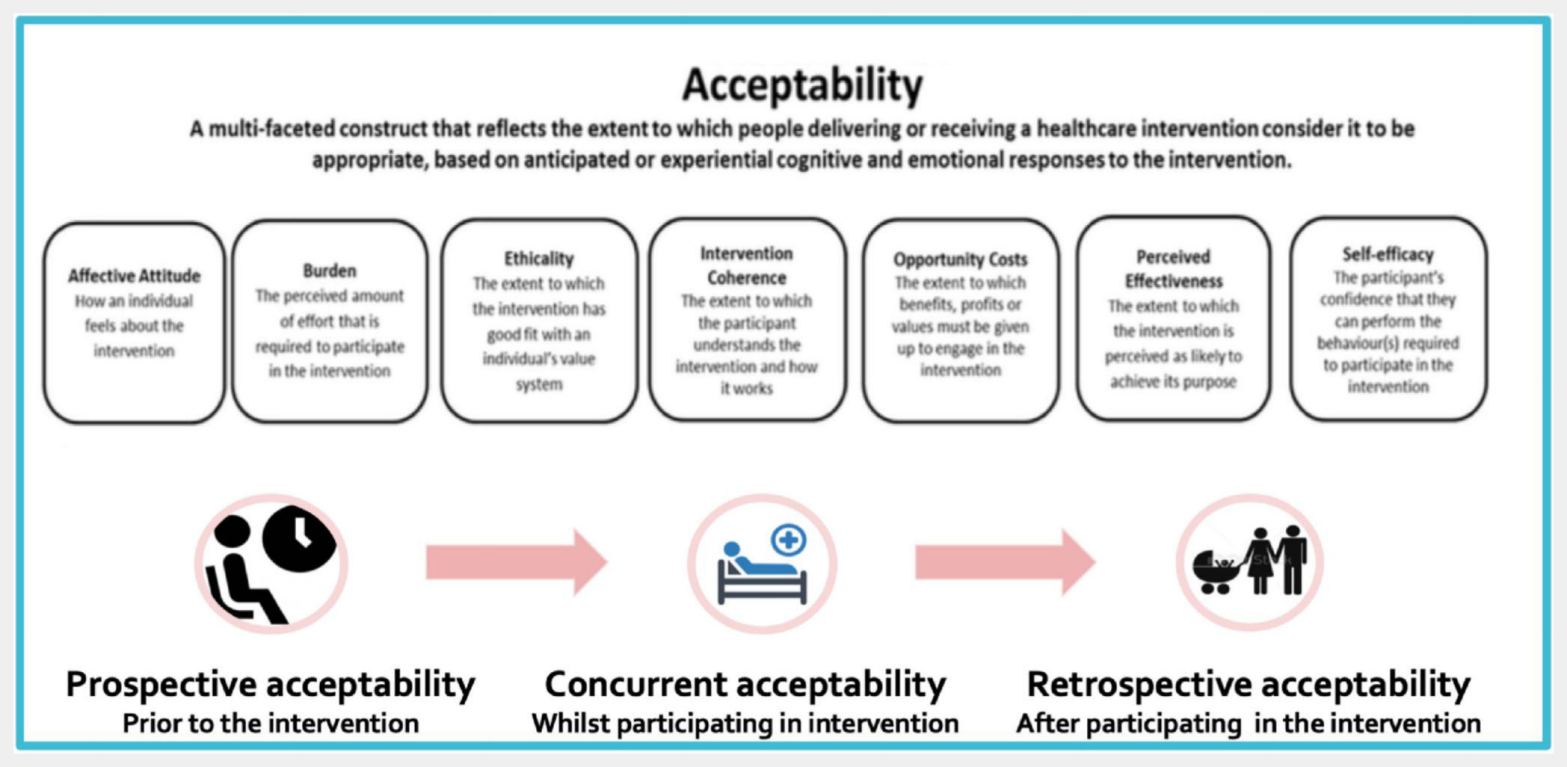
The framework of acceptability (adapted from the framework of acceptability by Sekhon et al.^12^) [Colour figure can be viewed at wileyonlinelibrary.com]

To assess the acceptability of MFS the Framework of Acceptability is used.^12^ It is the result of a systematic approach to how the acceptability of healthcare interventions should be defined, assessed and theorised. The framework has been applied to define and assess acceptability of healthcare interventions in a variety of healthcare settings.^13–16^ It comprised seven component constructs (Figure 1), that is affective attitude (AA), burden (B), ethicality (E), intervention coherence (IC), perceived effectiveness (PE), opportunity costs (OC), and self-efficacy (SE). The component constructs reflect the extent to which parents consider the intervention to be appropriate, based on cognitive as well as emotional responses to the intervention.^12^ As anticipated and actual lived experiences may differ, the framework measures acceptability over time: a) *prospective acceptability:* prior to participation in the intervention, b) *concurrent acceptability:* whilst in the intervention c) *retrospective acceptability:* after participating in the intervention.

### 2.2 Participant recruitment and selection

Participants were recruited at two MFS partner centres to ensure standardized specialist assessment (University Hospitals Leuven, Belgium; University College London Hospital, United Kingdom) between July 2018 and February 2021. Prospective parents that met the inclusion criteria for MFS, identical to those of the MOMS trial,^1^ were eligible to participate, irrespective of their subsequent decision. All eligible parents were informed about the study at the fetal medicine unit, and if interested their details were shared with the researcher (NC). Interested parents were contacted via email by the researcher (NC) with study information (Patient Information Sheet, Appendix 1a and b). Participants were excluded if they were under 18 years old or unable to speak English or Dutch. Women were given the option of whether their partner joined them in the interview or not. All participants gave written informed consent. For participants undergoing TOP one interview was planned; this was conducted at least 6 weeks post termination to avoid distress and emotional burden. For participants opting for MFS three interviews were planned: the first two interviews were undertaken face-to-face during hospital admission, one prior to MFS and one after the intervention during hospitalization. The third interview took place 3-6 months after the birth of the child, by video-phone or video-conference, unless parents requested an alternative. For participants opting for EM two interviews were planned: one during pregnancy after making their decision and one after the birth of their child. Recruitment ceased when saturation was reached.

### 2.3 Data collection

All interviews were conducted by an experienced researcher (NC) and started with an open question inviting participants to share their thoughts, views, feelings and experiences whether to participate or not in MFS.^17,18^ The seven framework components were used as prompts to further explore the different levels of the decision (Interview-guide, Appendix 2). Interviews lasted between 20 and 114 min, were audio-recorded and transcribed verbatim by a professional transcription service (LSD Business Services, Derbyshire, UK; Amberscript, Amsterdam, the Netherlands). Interview transcripts in Dutch were translated into English by NC for subsequent analysis.

### 2.4 Data analysis

Data analysis was performed using the Qualitative Analysis Guide of Leuven (QUAGOL)^19^. This method is characterized by the repetitive process and team approach. It consists of two parts, each with five analysis steps: the first aims to determine a conceptual understanding of the research data as a whole and the second comprises the coding process. Two researchers (AS and NC) read and re-read the interview transcripts, discussed observations and ideas, then formulated initial codes. Themes were identified and organised in line with the acceptability framework. Alternating between various stages of the process was required as new data and themes emerged, resulting in interaction between different parts of the analysis. The process was continued until data saturation was reached; no new information was obtained from subsequent interviews and thus recruitment was stopped.^20^ NVivo V12 software (QSR International Pty Ltd (2018)) facilitated data management, organisation and analysis. To enhance the rigour of the study the 32-item Consolidated Criteria for Reporting Qualitative research checklist was used (COREQ).^21^

### 2.5 Data presentation

**Figure 2:**
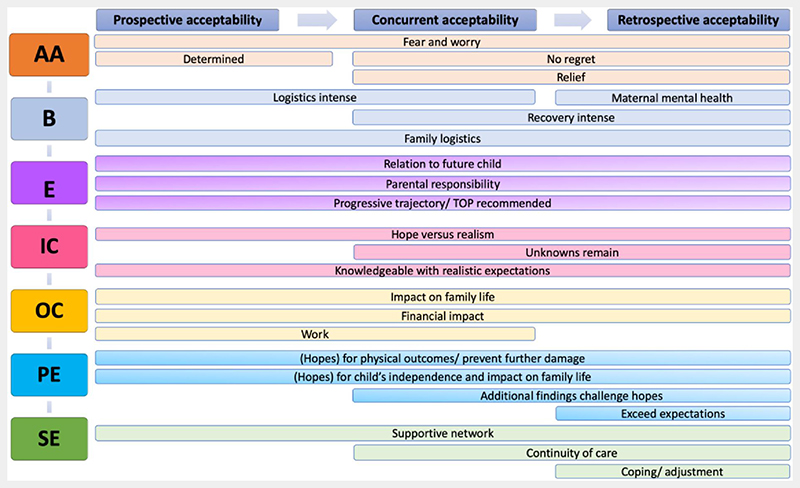
Identified themes of acceptability in temporal order, organized according to the theoretical framework of acceptability [Colour figure can be viewed at wileyonlinelibrary.com]

The themes derived from the interview analysis of parents opting for MFS are detailed in Figure 2, arranged in temporal order to indicate differences in acceptability over time, using the seven constructs identified by the framework of acceptability. Available results of the limited number of participants who opted for TOP and who were willing to participate, are presented descriptively. Themes extracted from the interviews with parents opting for MFS are illustrated by participants quotes, indicated in the text (e.g. Q1) and provided in Tables 1 to 3. Quotes are identified by father or mother and the patient’s number (1–28). The acceptability framework component construct relating to each theme is presented in parenthesis.

### 2.6 Ethics

This study was approved by the Ethics Committee of the University Hospitals Leuven, Belgium (S61586) and the South-Central Berkshire Research Ethics Committee UK National Research Ethics Committee (ref: 18/SC/0475).

## 3 Results

**Figure 3:**
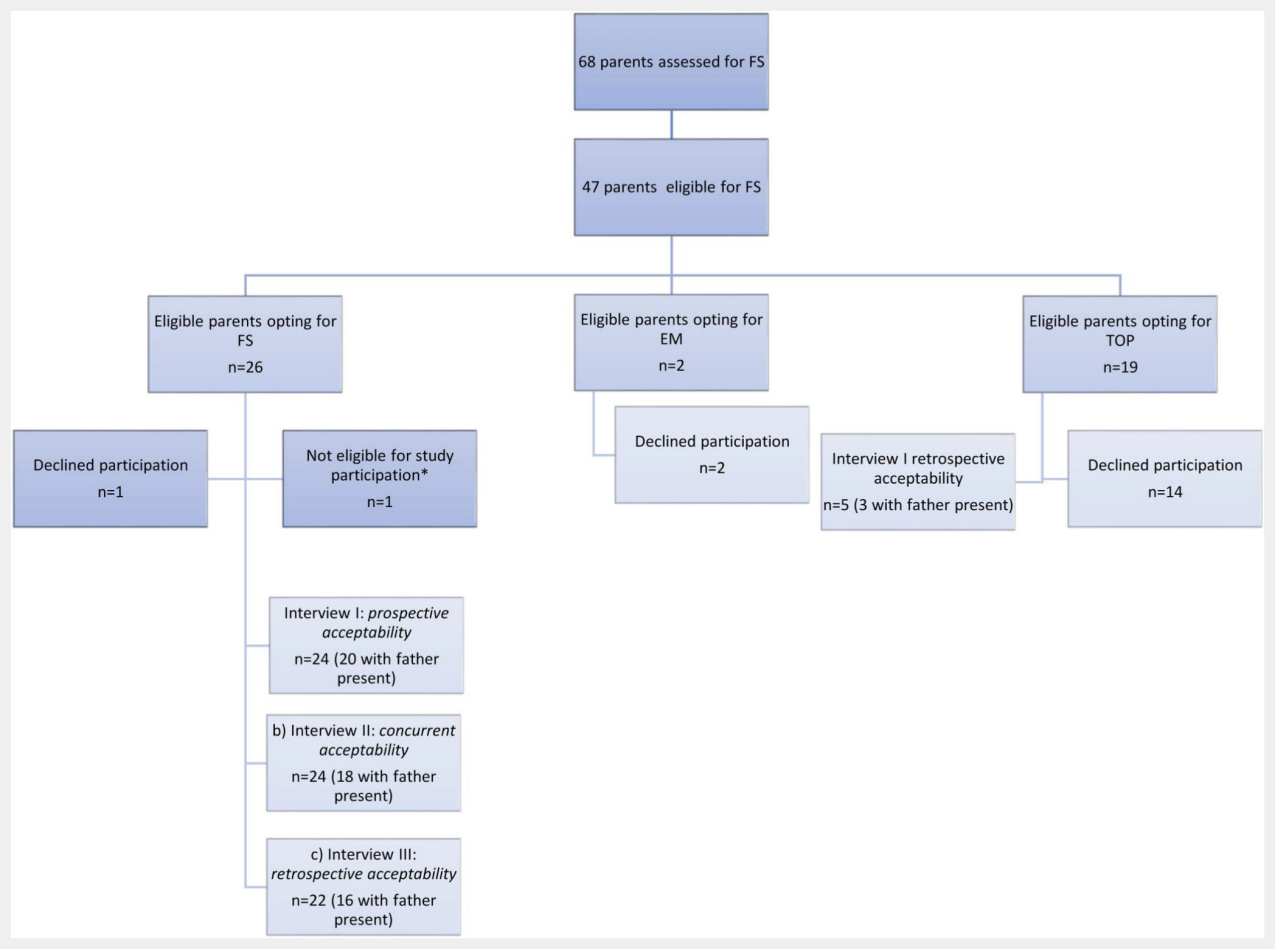
Assessed, eligible and included parents, per round of interviews (FS-fetal surgery; TOP-termination of pregnancy; EM-expectant management). *Unable to speak either Dutch or English [Colour figure can be viewed at wileyonlinelibrary.com]

Of 68 pregnant women with antenatally diagnosed fetal SB referred to the fetal surgical team over a 31-month period from July 2018 to February 2021, 47 were eligible for MFS. They were counselled about this option alongside potential alternatives including postnatal management or TOP. Of those, 26 opted for MFS, 19 for TOP and two for EM. Twenty-nine (62%) consented to study participation, with 24 parents opting for MFS (recruitment ceased October 2019 due to saturation) and only five (17%) opting for a TOP. No participants who were opting for postnatal surgery could be recruited to date (Figure 3). This paper reports the findings of the interviews with 24 participants (20 with partner present) who opted for MFS (Interview I: *prospective acceptability;* Interview II*; concurrent acceptability;* Interview III: *retrospective acceptability)* and provides perspectives of the five participants (3 with partner present) who opted for TOP (Interview I *retrospective acceptability*). Upon the request of both parents, one interview took place after their decision, but prior to TOP. The socio-demographic characteristics of the parents and neonatal outcomes are displayed in Table 4.

In total we conducted 75 interviews. Five with parents opting for TOP and 70 with parents opting for MFS. For the latter, interviews were conducted at three different time points: prior to the intervention (*n* = 24), during hospitalization (*n* = 24) and after birth (*n* = 22). Of the parents opting for MFS, two final interviews did not take place: one patient was lost to follow up and one mother who opted for neonatal palliative care following premature delivery declined further participation.

### 3.1 Attitudes and values

Parents opting for MFS actively chose to proceed with their pregnancy; for some because of the MFS itself however for most because they did not want to terminate the pregnancy. Parents reported strong feelings of parental responsibility and were determined to do anything to improve their future child’s health outcomes (E, Q1). Following successful MFS, parents expressed enormous relief. Firstly, because all went well, but moreover because they perceived MFS as an opportunity they were given (AA) to fulfil their perceived responsibility towards their unborn child’s wellbeing and outcome (E). The option of MFS provided hope for their child’s future, in contrast to the initial presentation of the condition as a hopeless perspective (E, Q2). Almost all parents opting for MSF perceived that TOP at diagnosis was the recommended option. After birth, parents either felt guilty at having considered TOP, or angry at their perception of being misinformed (E, Q16-18). None of the parents opting for MFS, regretted their initial decision (AA, Q9).

For the limited number of parents opting for TOP, the foreseen quality of life of their future child was decisive. SB was considered a condition impacting their future child’s quality of life to such an extent they felt it would be unfair to proceed with the pregnancy. For four parents, TOP was the only right decision, while one family weighed MFS against the option of TOP. Additionally, they considered the impact of a child with SB on (future) siblings and the family. MFS was perceived as providing too little certainty on the potential positive impact on their future child’s quality of life; maternal risks were not mentioned as reasons to decline. After the decision for TOP was taken, parents recalled ambivalent feelings of detachment from their fetus, often with the intention to protect their selves from too much grieving, coexisting with feelings of attachment towards their fetus, as well as intense grief.

### 3.2 Knowledge and risk perception

In general, all parents were knowledgeable and aware of the potential and substantial risks associated with surgery. For example, parents could recall the odds of their child being able to walk without surgery as well as the potential for improvement with the surgery. Parents opting for MFS were determined to proceed with surgery (AA, Q3) because it seemed to them the only option that could alter the natural course of the condition and potentially improve their child’s outcome (PE). Parents expressed hope that surgery would preserve motor functioning, but even more important, they hoped to preserve neuro-cognitive function (PE, Q4). Although driven by their hopes, potential complications and the uncertainty of surgical benefit made them fear their decision (IC).

The potential to increase the likelihood of their child being independent was perceived as a benefit to their family. In contrast, parents opting for TOP, did not consider MFS to add significantly to their future child’s outcomes and its quality of life. Risk related to the current pregnancy in both groups of parents were not considered a decisive reason to proceed or not with MFS. For parents opting for TOP, impact on potential future pregnancies was among the reasons to decline MFS.

### 3.3 Emotional impact

Shortly before having MFS women felt emotional and feared losing their unborn child. Some described a fear of not waking up after the operation, in particular those who already had children. Mothers termed this a ‘mother’s dilemma’: by trying to do the best for their unborn child they feared their own mortality, and even more that their other children would grow up without a mother (AA, Q6). For most parents, the main emotion after waking from their anaesthetic was relief (AA). Fathers reported their fears that they could lose both their partner and their unborn child, and described the intraoperative waiting time as stressful. This was even more pronounced when that period was longer than expected (AA, Q7). Facing hospital discharge, some parents feared a break in continuity of care up until the planned delivery date, as they had been monitored intensely throughout their stay (SE). They described the intensive monitoring as reassuring, and as a consequence the thought of being without it whilst at home prior to delivery was worrying (AA). Fear of premature birth was reported by some parents, whilst others felt confident all would be fine (AA). Parents opting for TOP expressed ambiguity: on one hand they indicated they would have wanted to care for this baby, but at the same time would feel selfish, if they would decide to put the burden of a disability on this child, just because they wanted to become parents. They grieved the loss of their (dreamed) child and some expressed feelings of guilt; all described it as an impossible choice they had to make.

Postnatally in the MFS group three mothers suffered from mental health issues following the birth of their child (B). One mother mentioned she was diagnosed with post-traumatic stress disorder and two mothers with depression; all had received treatment. Parents reported feeling the effects of all that had happened only after they got their baby home. One mother expressed extreme fears of losing her child that were triggered both at the moment of antenatal diagnosis as well as when the neonatal team indicated medical problems following her baby’s birth. She later experienced the same fears of death for her older child. Two further mothers reported a diagnosis of depression in the postnatal period, which they related to the distress they experienced from living through a “rollercoaster” that started at the moment of diagnosis (B, Q19-20). In the TOP group, three mothers and one father received bereavement support, which helped them to cope with their loss.

### 3.4 Burden and impact

Women opting for MFS discussed the logistical effort required to have MFS and reported significant and stressful difficulties organising last minute travel and accommodation (B). The stress of travelling and time away from work had a substantial financial impact on the families, and for some prompted the need to find additional funding for the MFS (B, Q5). Parents with children at home reported having to arrange care for their other children both during the hospital stay and recovery (OC).

The recovery in hospital, for some, was easier than anticipated but many mothers described the in-hospital recovery period as both physically and emotionally overwhelming. For some women the postoperative pain was described as extremely intense, however most women considered their hospital recovery as manageable due to the day-to-day improvements (B, Q8). Women experienced their home-based recovery very differently. Some were capable of doing some easy home and caring tasks, but others remained bed- or couch-ridden the whole pregnancy. One mother described so much pain following discharge she thought she and her baby were going to die (B, Q12). Hope for positive outcomes was the participants’ main drive to undergo the burden of the recovery process (PE).

### 3.5 Outcomes

At the final interview, all parents were happy with their child’s outcome which they reported as being often better than they hoped for (PE). For some parents, however, certain outcomes such as hip-dislocation, despite being diagnosed, was disappointing or unexpected. Along with any additional unexpected findings such as reasons for longer hospitalization or not meeting certain milestones, challenged parents’ hopes (PE, Q14). All parents discussed that there remained many uncertainties and acknowledged that the child’s outcome would be revealed in time (IC, Q15).

### 3.6 Continuity of care

Following MFS, a substantial proportion of women reported a break in the continuity of their pregnancy care, caused by what they perceived as lack of knowledge or understanding about their situation amongst their routine antenatal healthcare providers (SE, Q10). Some parents reported their concerns were not taken seriously by local clinical staff, which exacerbated their worries (AA, Q11) and made them feel responsible for their unborn child’s wellbeing and outcome (SE). These concerns continued into the neonatal period, where parents reported a perceived lack of understanding or knowledge of their situation amongst the neonatal staff, giving them a feeling of not being taken seriously. Parents worried these challenges could offset all the investments they had made so far for their child, reporting a perception that they needed to provide extra protection for their newborn (E, Q13).

## 4 Discussion

In this cohort of parents, acceptability for MFS was assessed by using the acceptability framework. For parents opting for MFS, acceptability remained high at each of the three interviews undertaken throughout their journey. Prior to the intervention it was perceived as an opportunity that needed to be taken. Parents seemed well aware of the risks involved and the uncertainty about individual outcomes. Expectations seemed to be realistic yet were driven by hope and expectation of the best outcome. None expressed regrets about their decision at any stage, despite the substantial impact. Few parents who opted for TOP were willing to participate. Those who did, considered the severe impact of SB on the quality of life of their future child as decisive. For most, TOP was the preferred option, one family weighed MFS against the option of TOP, but concluded that it would not add substantially to improve the quality of their future child’s life.

Earlier studies providing an insight into parental experiences about MFS for SB confirm the high acceptability of the intervention. Most parents do not express regrets about their decision^22^ and hold positive attitudes towards MFS.^23^ Among parents opting for MFS, parental-fetal attachment, in this study described as the perceived parental responsibility towards their unborn child, is a construct that indicates a unidirectional and ‘abstract’ relation of the parents towards their fetus.^24–26^ This seems an important motivating factor in the parents’ decision to undertake MFS. Studies have indicated an increase in this phenomenon among parents receiving a prenatal diagnosis of a serious congenital malformation, including surgical conditions and twin-to-twin transfusion syndrome.^27,28^ In contrast, parents opting for TOP express this parental responsibility in the context of protecting their child from a life with significant challenges and MFS was not considered to improve this significantly.

Many parents in this study recalled a strong contrast between the initial prenatal diagnosis and the subsequent option of MFS. MFS was perceived as an opportunity for potential improvement of their baby’s outcome, giving hope for a more positive future outlook. Some parents even experienced feelings of guilt for having considered TOP. This memory remained powerful, and parents often reflected on it, both after surgery and the birth of their child, a finding also seen in mothers who have experienced severe congenital heart defect in their unborn child.^29^ The parents in this study valued information and care focusing on the future from the healthcare providers, and the recognition of the fetus as a future child, similar to prenatal diagnostic trajectories of parents of children with Down syndrome.^30–32^ A perceived break in the continuity of care between treatment centre and referral centre caused additional stress to an already intense trajectory. Families often are referred long-distance, or even international, which stresses the importance of a smooth transition and demands clear communication between centres.

The observation of attachment to their unborn child is interesting in the context of the debate on whether a fetus should be considered as a patient.^33,34^ Our interviews make it clear that the study participants already perceived their fetus as their future child from the moment they entered the MFS trajectory, and is similar to parents confronted with antenatal diagnosis of twin-to-twin transfusion syndrome, early onset fetal growth restriction and fetal congenital cardiac defects.^27,28,35^ In contrast, in the TOP group, parents grieve the loss of their (dreamed) child, which they have tried to protect from a life with additional challenges, leading to coexistent feelings of detachment and attachment, which are known in comparable groups of parents.^36^

Three out of 24 mothers who opted for MFS were diagnosed with mental health issues after birth of their baby, which they relate to events they experienced during their pregnancy trajectory. Earlier studies have reported depression and anxiety in patients undergoing fetal diagnosis or receiving fetal therapy, suggesting that women underdoing MFS require appropriate follow-up and support to detect increased risks of mood and anxiety disorders.^37–39^ This was already the case in women with fetal SB in the pre-fetal surgery era,^40,41^ to which the stress of the MFS and potential complications of prematurity is now added.

In this cohort, most parents, including the three mothers that were diagnosed with mental health issues, reported adjusting adequately to their new situation, regardless of the individual outcome. Hope, parenting satisfaction, income, marital adjustment, economical resources and (social) support are factors improving parental psychological adjustment in parents of children with SB, in contrast to disability related stress, negative family impact and negative coping strategies.^41^ In this cohort, parental hopes, parenting satisfaction, and their social support network may have positively contributed to this early adjustment. Participants also reported that they felt adequately supported by the team’s counselling and interaction, and even by study participation itself, the latter has been confirmed by others as well.^42–44^

### 4.1 Strengths and limitations

One strength of this study is the high participation rate of parents who opted for MFS (24/26) in the period under study. A diverse case mix was seen, with heterogeneity for parity, age and educational level, enhancing the generalisability. We assessed their experience against a framework of acceptability using previously validated methodology. Another strength is that this study provides a first in-depth exploration of parental experience of fetal surgical SB repair focussing on the acceptability of the intervention, and thereby supports the provision of patient-centred care.^45–47^ Two recent studies describing the information recommended to counsel parents whose fetus has SB did not address the patient perspective, or did so to a limited extent.^48,49^ However, this study has several limitations. We only had access to women referred for potential surgery at two centres managed by identical surgical teams. This ensured that parents received standardized specialist assessment and counselling regarding all three options. Patients who were referred were more likely to opt for MFS. This may explain the lower number than expected of parents opting for TOP and even no inclusions in the group of parents choosing postnatal surgery. Therefore, we are actually only able to provide some insights into these populations. We acknowledge the importance of the contrasting parental perspectives on providing a full scope and balanced presentation of what defines the acceptability of MFS to all parents. Given the relevance of different parental perspectives, further recruitment for TOP and EM should go on. Lastly, one mother experienced a neonatal loss. Due to persisting grief, this mother preferred not to participate in the final (postnatal) interview, therefore her perception of acceptability of what she was offered has not been explored.

## 5 Conclusion

MFS for SB remains highly acceptable from diagnosis until 3-6 months postnatally. In contrast, parents who opted for TOP, MFS was not perceived as an intervention that substantially could improve the quality of their future child’s life. For those opting for MFS, expectations seemed to be realistic yet were driven by hope and expectation of the best outcome. For parents opting for TOP, the potential benefit of MFS seems to play a minimal role in their final decision.

## Supplementary Material

Additional supporting information may be found online in the Supporting Information section at the end of this article.

Supplementary Information

## Figures and Tables

**Table 1:** Prospective acceptability, example quotes

Theme	Example Quote
Ethicality (E)	Q1 “If it doesn’t work, if he needs a shunt and he never walks, well at least we can say we did everything we could at the time that was available. And you can look him in the eyes and say, ‘look, we tried anywayş’.” *Father12*
	Q2 “But it was kind of a progression thing, that first meeting was awful and you know that second meeting was more beneficial, we were talking to more specialized individuals, that’s when we talked to the neurosurgeon and then the specialist in the delivery.” *Father2*
Affective attitude (AA)	Q3 “But we’ve come to the decision now and it’s not something that, yes, I’m terrified of it, but I wouldn’t go back on it, at all. You know, and if in the morning, if they told me I couldn’t do it, I think it would devastate me, because this is the decision we’ve come to.” *Mother17*
Perceived effectiveness (PE)	Q4 “It sounds a bit strange, but psychologically for me, just to know every day you’re kind of waking up waiting for the kicks, is that a kick? Is that a punch? […] so if this back is closed from 26 weeks, just to be able to relax a little. I’m not saying I’m going to enjoy the pregnancy but just to know that, right, no more damage can be done, that’s a big thing.” *Mother12*
Burden (B)	Q5 “I think a couple of times we kind of went ‘do we have enough [money]?’ But I suppose in the long run, it’s worth it. Yeah, I mean, as [mother] said, we’ve got family support, so if we do need money, they’re willing to give it.” *Father16*

**Table 2:** Concurrent acceptability, example quotes

Theme	Example Quote
Affective attitude (AA)	Q6 “Well, it’s actually all about the children you’ve got and then the child that you’re trying to protect at that time. So, there’s not really a right one to go by for a mother.” *Mother24*
	Q7 “That’s one time where – I forgot about that – that is the one time where I thought I was going to lose it is when she was in prenatal surgery and nobody told me that she was out of prenatal surgery.” Father2
Burden (B)	Q8 “And I think it’s a good thing to have, to be honest, because I think it’s healthy where I was just like, ‘why have I done this? I Feel awful. I don’t ever want to do this again. I Never want — I’m just suffering so much, why put myself through this? Why couldn’t I just let it go to natural causes and just see what happens?’ there was definitely that element there for a day, definitely, which was my lowest point.” *Mother10*
Affective attitude (AA)	Q9 “I could turn around to somebody now, honestly, hand on my heart, and say, ‘do you know what? If I had to do it again, I would’.” *Mother15*

**Table 3:** Retrospective acceptability, example quotes

Theme	Example Quote
Self-Efficacy (SE)	Q10 “Every time we were going for check-ups there was a different doctor checking her. And different doctors were putting different notes. and like towards the end, maybe a month before the c-section, there was a doctor and she wrote the abbreviation for natural birth: NB.” *Father22*
Affective attitude (AA)	Q11 “Because any delivery in general pregnancy is not easy, even if it’s normal. Every woman has something else. But this was - I’m not saying I’m the only one with a child with spina bifida with the surgery and everything, but it’s not a normal pregnancy, it’s hard on its own, you know.” *Mother22*
Burden (B)	Q12 “The week before I had her, I actually thought we were both going to die, I was in that much pain […] yeah, they underestimated how I felt. But it was that bad I didn’t think we were going to make it. Yeah, it was horrible.” *Mother11*
Ethicality (E)	Q13 “I wanted [to go] home because you felt like you were playing doctor as well as mummy, when really all you needed was to be playing mum with a wee bit of support. […] I don’t know if it’s because his surgery was done [elsewhere] before birth, so when he was born, they just weren’t sure where he slotted in on the timeline of what should be happening when. […] because he was kind of nine weeks past his surgery, they were like, what do you do? What do you? Does he need all this?” *Mother15*
Perceived effectiveness (PE)	Q14 “Yeah, I wouldn’t say I regret the surgery, I think it’s just disappointing that she does have the hip problem because we weren’t expecting it.” *Mother5*
Intervention coherence (IC)	Q15 “.we’ll just have to see, but because we’re so happy with her and we’re in a bubble, we don’t think there’s anything wrong so I guess if one day they said, you know, she’s going to need a wheelchair, she’ll need crutches, of course it will be heart-breaking but we - these are the good years because we do everything for her.” *Mother20*
Ethicality (E)	Q16 “Then it hits that you that at one point we were sort of heading towards her not being here. So that hits you quite often.” *Mother3*
	Q17 “They said to us, [at diagnosis], yes, your child is handicapped, it will be in a wheelchair and [euh] she may have brain damage, and those things. It was brought as a very dark outlook. [..] but now, if, in hindsight, if I think, no, how have I ever been able to question this decision [of fetal surgery]. I Should not have questioned it...But it is a lack of knowledge.” *Mother19*
	Q18 “Then once she came out, obviously her legs were kicking and then she wee’d everywhere. They were like, ‘oh, she’s not going to do any of this,’ and I was like, do you know what? Stuff the world. I said, ‘look, she’s come out doing everything they said she wouldn’t, and their idea of an abortion is her’.” *Mother7*
Burden (B)	Q19 “Yes, it does, and then when everything starts to settle and you’ve had time in the house and time to actually think about things, you realise look what we’ve just been through. You know, it hits you then like a train and you’re like oh my goodness.” *Mother15*
	Q20 “It happened at the 20 weeks anomaly scan [diagnosis of spina bifida]. Then I really thought this baby is not going to come……And then, after she was born, they called me to get down to the neonatal ward, again I thought [name of child] is going to die, you know? Those moments were just very intense….and at a certain moment….I envisioned that [first child] was in the water, drowned. All those things. Just an enormous fear to lose both my kids.” *Mother19*

**Table 4:** Socio-demographic characteristics of study participants

	Maternal-Fetal Surgery		Termination of Pregnancy	
	Mothers *n* (%) 24	Fathers *n* (%) 22^a^	Mothers *n* (%) 5	Fathers *n* (%) 3
Age				
20–29 years	9 (38%)	4 (18%)	1 (20%)	–
30–39 years	15 (62%)	14 (64%)	3 (60%)	3 (100%)
>40	0 (0%)	4 (18%)	1 (20%)	–
Level of education				
Low	4 (17%)	5 (23%)	–	–
Intermediate	3 (12%)	6 (27%)	1 (20%)	1 (33%)
High	17 (71%)	11 (50%)	4 (80%)	2 (67%)
Parity				
Nulliparity		7 (29%)		2 (40%)
Multiparity		17 (71%)		3 (60%)
Marital status				
Partner		23 (96%)		5 (100%)
Single		1 (4%)		–
Country of origin mother				
United Kingdom and crown dependencies		12 (50%)		
United States of America		1 (4%)		–
Ireland		3 (13%)		–
The Netherlands		3 (13%)		1 (20%)
France		1 (4%)		1 (20%)
Croatia		1 (4%)		–
Hungary		1 (4%)		–
Hong Kong		1 (4%)		–
Finland		1 (4%)		–
Belgium		–		3 (60%)
Country currently resident				
United Kingdom and crown dependencies		15 (63%)		–
Ireland		5 (21%)		–
The Netherlands		4 (17%)		1 (20%)
Belgium		–		4 (80%)
Treatment centre				
Belgium (UZ leuven)		19 (79%)		5 (100%)
United Kingdom (UCLH)		5 (21%)		–
Gestational age at birth				
<24 weeks		–		5 (100%)
25–28 weeks		1 (4%)		–
29–32 weeks		1 (4%)		–
33–36 weeks		13 (54%)		–
>36		9 (38%)		–

a Data from one father missing.

## Data Availability

The data that support the findings of this study are available on request with reasonable motivation from the corresponding author. The data are not publicly available due to privacy or ethical restrictions.

## Key points

### What’s already known about this topic?

- Maternal-fetal surgery for open spina bifida has the potential to improve long-term outcomes but at significant procedure-related fetal and maternal risk;
- Prospective parents receiving an antenatal diagnosis of open spina bifida, face a range of uncertainties regarding the future of their unborn child, and the options provided pose major ethical challenges.

### What does this study add?

- Despite significant impact, maternal-fetal surgery is highly acceptable for those parents who opt for it; Strong feelings of parental responsibility, direct them to do anything in their power to improve their future child’s situation;
- In the small group of parents who opted for termination of pregnancy, maternal-fetal surgery was felt to offer insufficient certainty of substantial improvement in quality of life and the perceived severe impact of SB drove their decision to end the pregnancy.
