# Supplementary material for: ‘We did everything we could’– a qualitative study exploring the acceptability of maternal-fetal surgery for spina bifida to parents
Source: Prenat Diagn. Author manuscript; Available in PMC 2022 Sep 7. (PMC7613560; doi:10.1002/pd.5996)
Supplement: Supplementary Information [file EMS152847-supplement-Supplementary_Information.zip › pd5996-sup-0001-suppl-data.pdf]

GIFT Surg
Guided Instrumentation for Fetal Therapy and Surgery

## Participant Information Sheet

Version: 2.0 Date: 9/OCTOBER/2018 REC Reference: 18/SC/0475

Principal Investigator: Professor Neil Marlow, Consultant in Neonatal Medicine
Researchers: Neeltje Crombag PhD, Midwife and Research Fellow,
Professor Anna David, Consultant in Fetal Medicine

# Fetal surgery interview study: Parental perceptions of Fetal Surgery

You are being invited to take part in an interview study. Before you decide, we would like you to understand why the interviews are being done and what it involves. Please take time to read the following information carefully and discuss it with others if you wish. Your decision will be respected and will not affect the standard of care you are receiving. One of our team will go through the information sheet with you and will answer any questions.

**What is the purpose of the study?**

During this pregnancy, your baby has been diagnosed with spina bifida. As you might know these are problems that often arise early in pregnancy and with a large variety of possible effects on your child's health. You will have spoken with a Doctor and you have had the chance to obtain all of the information that you need about your baby's diagnosis and about all of the choices available to you. When your baby is diagnosed with spina bifida, there are three options: postnatal therapy, termination of pregnancy or the option for fetal surgery. Fetal surgery, is a surgical procedure carried on a baby before it is born. This study is part of a larger research project, called Guided Instrumentation for Fetal Therapy and Surgery (GIFT-Surg), which aims to improve the results of fetal surgery, primarily for these two conditions.

As surgery on the baby before it is born is a very new treatment, we are interested in the factors that influence your decision about what to do in this pregnancy, whether or not you decide to proceed with the pregnancy or choose termination, or choose fetal surgery or surgery after birth. So that we can help to design the support we give to parents in these situations, we would like to find out how you view these difficult decisions, and what the thoughts, feelings and needs are of parents experiencing this stressful period. We call this the parental perspective. Learning more about your perspective will help us to improve support and care for future parents facing the same difficult decisions.

**Why have I been invited?**

You have been invited in this study as your baby has been diagnosed with spina bifida and you are able to choose the option of fetal surgery. We are interested in all parents facing the dilemmas when expecting a child with likely spina bifida or diaphragmatic hernia, regardless of any decision you have made or will make.

**Do I have to take part?**

No. It is up to you to decide to be interviewed and your decision will be respected. If you agree to be interviewed, we will ask you to sign a consent form. You are free to withdraw at any time, without giving a reason. If you would rather not take part, or if you later want to withdraw, this will not affect the care you receive.

**What happens if I am interested in taking part?**

You may discuss this with the member of the team who discussed the project with you or contact Neeltje Crombag. Dr Crombag is a midwife and research fellow employed by the GIFT-Surg study specifically to carry out these interviews. She will arrange a time to speak to you on the telephone to go over the details of the interview study and answer any of your questions, which should not take more than 20 minutes.

**Can my partner take part in the interview?**

Yes. Please let us know when you contact us whether your partner would like to take part in the interview. If possible we would like to speak to both of you before the interview.

**What will I have to do if I decide to take part?**

We would like to have two or three face-to-face interviews, with you and your partner. One interview after your first consultation, and one, three months after the birth of the baby, or if you choose to terminate the pregnancy, three months after the termination. If you decide to have fetal surgery, one additional interview will take place during the week of hospitalisation after the intervention. These interviews will all take place alongside a hospital visit, while you are hospitalised and/or via Skype or phone. It will last approximately 30-60 minutes and will be recorded as an audio-file. During the interview, we will ask you to share your thoughts, views, feelings and experiences related to the decision about what to do in this pregnancy. As we are interested in the individual experiences of expecting parents, there are no right or wrong answers, and we would like to learn about your thoughts and experiences.

**What are the possible inconveniences and risks of taking part?**

It may be distressing for you to speak about your experience in this pregnancy, in which you have heard the news your baby is affected by spina bifida. You will also need to speak to us on the telephone for about 20 minutes to discuss the study and arrange for the interviews. The first interview will be face-to face but combined with a scheduled hospital visit. Subsequent interviews can be done via Skype or phone upon your preference.

**What are the possible benefits of taking part?**

There is no immediate and direct benefit to you. However, by taking part in the study you are supporting research into fetal therapy, and in particular care for future parents who also have a pregnancy affected by the same and similar conditions. For some parents sharing their feelings, experiences and thoughts helps them to cope with the stressful period they go through.

**Is there any payment for taking part?**

You will receive expenses when you need to travel for the study, but no other payments for taking part.

**Will my taking part in the study be kept confidential?**

The interview will be audio-recorded and transcribed for the purpose of analysis. Transcription will be carried out by a professional transcription service, and all information will be anonymised so neither you nor your doctors can be recognised from the written transcript. All information about you will be kept confidential, following legal and ethical practice, unless there is serious risk of harm. No information will be disclosed outside the study without first discussing this with you.

**What will happen to information about me?**

UCL is the sponsor for this study based in London, the United Kingdom. We will be using information from you and/or your medical records in order to undertake this study and will act as the data controller for this study. This means that we are responsible for looking after your information and using it properly. UCL will keep identifiable information about you for 2 years after the study has finished.

Your rights to access, change or move your information are limited, as we need to manage your information in specific ways in order for the research to be reliable and accurate. If you withdraw from the study, we will keep the information about you that we have already obtained. To safeguard your rights, we will use the minimum personally-identifiable information possible.

You can find out more about how we use your information at https://www.ucl.ac.uk/legal-services/data-protection-overview, or by e-mailing the UCL Data Protection Office [data-protection@ucl.ac.uk].

Any information that can be used to identify you (name, address, telephone number) will be stored securely at UCL, the university that is sponsoring the study, either in a locked filing cabinet or on a securely protected computer. The recording of the interview will be given a code/identification number and will not include your name or contact details. The recording, a written version of the interview and the information we get from these will be stored securely at UCL on a password protected computer. Information about you may be looked at by authorised staff only: Dr Crombag and Professor Neil Marlow. Both have a duty of confidentiality to you. Your personal details will only be kept whilst we need to contact you, following which they will be securely destroyed. Recordings and the written versions will be kept for 2 years after the study ends. There will be nothing that identifies you in this material or in the results.

UCL will use your name and contact details to contact you about the research study, and make sure that relevant information about the study is recorded for your care, to oversee the quality of the study. Individuals from UCLH and regulatory organisations may look at your medical and research records to check the accuracy of the research study. UCLH will pass these details to UCL along with the information collected from you and/or your medical records. The only people in UCL who will have access to information that identifies you will be people who need to contact you to audit the data collection process. The people who receive the results of the study will not be able to identify you and will not be able to find out your name, NHS number or contact details.

UCL will keep identifiable information about you from this study for 2 years after the study has finished.

**What will happen if I don't want to carry on in the study?**

You can withdraw from the interview at any time by contacting the researcher.

**What if I find the interview upsetting?**

You can contact our local Fetal Medicine Unit midwives or your GP. You may also find it useful to contact the following charities: Shine charity at www.shinecharity.org.uk or 01733555988 or firstcontact@shinecharity.org.uk, ARC (Antenatal Results and Choices) at www.arc-uk.org or 0845 077 2290; BLISS at www.bliss.org.uk or 0500 618140; SANDS at www.uk-sands.org or 020 7436 5881.

**What if there is a problem?**

Please ask to speak to the researchers who will answer your questions (neeltje.crombag@kuleuven.be or a.david@ucl.ac.uk). If your problem is not resolved or you want to speak to someone independent you can contact the UCLH Fetal Medicine Unit on 020 3447 6195 If you remain unhappy and wish to complain formally, you can contact Patient Advisory Liaison Services (PALS) on 020 3447 3042.

**Who is organising and funding the research?**

The study is being run by the University College London Hospital, is sponsored by University College London and is funded by the Wellcome Trust and the Engineering and Physical Sciences Research Council (EPSRC).

**Who has reviewed the study?**

All research in the NHS is looked at by two independent groups of people. All patient-based research is reviewed first by the Health Research Authority, and subsequently by one of their Research Ethics Committees, to protect your interests. This study has been given favourable opinion by South Central - Berkshire Research Ethics Committee.

**What will happen to the results of the research study?**

The findings will be published in medical journals and a summary will be sent to you, if you so wish, and will be available on the GIFT-Surg website (www.gift-surg.ac.uk). You will not be identified in any report or publication, but we may include anonymous direct quotations from the interview.
