# Supplementary material for: ‘We did everything we could’– a qualitative study exploring the acceptability of maternal-fetal surgery for spina bifida to parents
Source: Prenat Diagn. Author manuscript; Available in PMC 2022 Sep 7. (PMC7613560; doi:10.1002/pd.5996)
Supplement: Supplementary Information [file EMS152847-supplement-Supplementary_Information.zip › pd5996-sup-0002-suppl-data.pdf]

Title of the study: The parental perspectives in decision-making for fetal surgery

Sponsor: *University Hospitals Leuven*

*Department: Development and Regeneration, Biomedical Sciences, Herestraat 49, 3000 Leuven*

Ethical committee: *Ethical Committee Research UZ/ KU Leuven.*

Study supervisor and researchers: *Prof. Jan Deprest, jan.deprest@uzleuven.be, Fetal Medicine, UZ Gasthuisberg, Herestraat 49, bus 7003 06, 3000 Leuven; researcher: Neeltje Crombag neeltje.crombag@kuleuven.be*

## I Important information for deciding whether to take part

### Introduction

You are being invited to take part in an interview study to investigate the parental perspectives in pregnancies where fetal surgery is an option. Before you decide, we would like you to understand why the interviews are being done and what they involve. Please take time to read the following information carefully and discuss it with others if you wish. Your decision will be respected and will not affect the standard of care you receive. One of our team will go through the information sheet with you and will answer any questions.

Before you agree to take part in this study, we would like you to be aware of the time commitment, possible disadvantages and benefits, to allow you to make a decision with full awareness of the implications. This is known as giving "informed consent".

Please read this information carefully and ask the investigator (Prof Jan Deprest) or his representative any questions you have. There are three parts to this document: the information essential to your decision, your written consent and supplementary information (appendices) detailing certain aspects of the study.

### If you take part in this clinical study, you should be aware that:

- This clinical study is being conducted after review by one or more ethics committees.
- Your participation is voluntary and must remain free from any coercion. It requires the signature of a document expressing your consent. Even after signing this document, you can stop taking part by informing the investigator. Your decision not to take part or to stop taking part in the study will have no impact on the quality of your care or on your relationship with the investigator.
- The data collected is confidential and your anonymity is guaranteed during publication of the results.
- Insurance has been taken out in case you should suffer any damage in connection with your participation in this clinical study.
- You will not incur any charges for the visits/consultations, examinations or treatments specific to this study.
- You may contact the investigator or a member of his team at any time should you need any additional information.

Further information about your "Rights as a participant in a clinical study" can be found on page 5.

### Objectives and description of the study protocol

During this pregnancy, your baby has been diagnosed with a structural (physical) anomaly (abnormality) and fetal surgery is among the options. As you might know, structural anomalies are often detected in pregnancy and have a large variety of possible effects on the future child's health.

You will have hopefully spoken with an expert and had the chance to obtain all of the information that you need about your baby's diagnosis and about all of the choices available to you. Among other options, such as postnatal surgery or termination of pregnancy, there is the option for fetal surgery. Fetal surgery is a procedure carried out on a baby before it is born. We are inviting you to take part in an interview study to investigate the parental perspectives in pregnancies in which fetal surgery is among one of the options. This study is part of a larger research project, called GIFT-Surg, which aims to improve the results of fetal surgery, primarily for spina bifida and congenital diaphragmatic hernia. As surgery on the baby before it is born is a new treatment, we are interested in the factors that influence your decision about what to do in this pregnancy, *whether or not you decide to proceed with the pregnancy or to take up the option of fetal surgery*. So that we can improve the support we give to parents in these situations, we would like to find out how *you* view these difficult decisions, and what *your* thoughts, feelings and needs are during this stressful period. We call this **the parental perspective**. Learning more about your perspective will help us to improve support and care for future parents facing the same difficult decisions.

## Course of the study

We would like to have a face-to-face interview, alongside a hospital visit, either with or without your partner. The interview will be carried out by a midwife and research fellow, Mrs Neeltje Crombag. She is experienced both in carrying out these types of interviews, and is experienced in caring for patients in similar situations. During the interview, we will ask you to share your thoughts, views, feelings and experiences related to the decision about what to do in this pregnancy. As we are interested in the individual experiences of expecting parents, there are no right or wrong answers, and we would like to learn about *your thoughts and experiences*.

If you decide to take part in the study, you will be invited for at least two interviews: the first interview will take place shortly after one of your hospital consultations. The second interview will take place several months after the birth of the baby, or termination of the pregnancy. This will be scheduled according to your preference and availability, either by Skype or phone. If you decide to have fetal surgery, a third interview will take place after the surgery, while you are still in the hospital and feel well enough to speak with the researcher.

The interview will last approximately 30-60 minutes, in which the interviewer will mainly listen to your personal experiences. The interviews will be recorded as an audio-file, and transcribed verbatim (word-for-word) to facilitate the subsequent analysis.

Any information that can be used to identify you (name, address, telephone number) will be stored securely at UZ Leuven, the hospital that is sponsoring the study, either in a locked filing cabinet or on a securely protected computer. The recording of the interview will be deleted from the devices and computer, after the transcription and accuracy check. The written version of the interview and the information we get from these will be stored securely at UZ Leuven servers, on a password protected computer. Information about you may be looked at by authorised staff only: Mrs Crombag and Professor Jan Deprest. Both have a duty of confidentiality to you. Your personal details will only be kept whilst we need to contact you, following which they will be securely destroyed. The written versions will be kept for 20 years after the study ends. There will be nothing that identifies you in this material or in the results.

## Benefits and disadvantages

*Possible benefits:*

There is no immediate and direct benefit to you. However, by taking part in the study you are supporting research into fetal therapy, and in particular, care for future parents who also have a pregnancy affected by similar conditions. For some parents sharing their feelings, experiences and thoughts helps them to cope with the stressful period they go through.

*Possible disadvantages:*
You will need to speak to us prior to the interview, for about 20 minutes to discuss the study and arrange for the interviews. The first interview will be face-to face but combined with a scheduled hospital visit and lasts 30-60 minutes. Subsequent interviews can be done via Skype or phone upon your preference.

**Withdrawal from the study**
Your participation is voluntary and you are entitled to withdraw from the study for any reason, without having to justify your decision. Nevertheless, it may be useful for the investigator and for the sponsor of the study to know if you are withdrawing because the demands of participation are too great.

**If you take part in this clinical study, we ask you to:**

- Help us in the smooth running of this study.
- Be present at the scheduled timing of the interview and to participate in the interview
- Inform the researcher if you consider participating in another study, to discuss whether you can participate in both studies or not

When the study is finished, you will receive a summary of the results.

**Contact**
If you need additional information or if you have any concerns you can contact the investigator (Professor Jan Deprest) by phone +32 16 34 51 23 (working hours) or e-mail (Jan.Deprest@uzleuven.be) or a member of his research team (Neeltje Crombag, neeltje.crombag@kuleuven.be, by phone: +32 16345222 who will answer your questions or contact the study supervisor.

If you have any questions relating to your rights as a participant in a clinical study, you can contact the patient rights ombudsman of UZ Leuven on this telephone number +3216 34 48 18. If necessary, he/she can put you in contact with the ethics committee.

Title of the study: The parental perspectives in decision-making for fetal surgery

## II Informed consent

### Participant

I declare that I have been informed of the nature of the study, its purpose, its duration, any risks and benefits and what is expected of me. I have taken note of the information document and the appendices to this document.

I have had sufficient time to think about it and discuss it with a person of my choice, such as my GP or a member of my family.

I have had the opportunity to ask any questions that came to mind and have obtained a satisfactory response to my questions.

I understand that my participation in this study is voluntary and that I am free to end my participation in this study without this affecting my relationship with the therapeutic team in charge of my health.

I understand that data about me will be collected throughout my participation in this study and that the investigator and the sponsor of the study will guarantee the confidentiality of these data.

I agree to my personal data being processed as described in the section dealing with confidentiality guarantees (page 5). I also consent to these data being transferred to and processed in countries other than Belgium.

***I agree/ I do not agree*** (delete as appropriate) to the study data being processed at a later date, provided this processing is within the context of the present study, for a better understanding of the disease and its treatment or improvements in patient communication and care.

I have received a copy of the information to the participant and the informed consent form.

*Surname:* …………………………………………… *First name:* ……………………………………………

*Date:* ……………………………………………

*Signature of the volunteer:* ……………………………………………

**Investigator**

I, the undersigned, [surname, first name] investigator/clinical study assistant, confirm that I have verbally provided the necessary information about the study and have given the participant a copy of the information document.

I confirm that no pressure was applied to persuade the patient to agree to take part in the study and that I am willing to answer any additional questions if required.

I confirm that I operate in accordance with the ethical principles set out in the latest version of the "Helsinki Declaration", the "Good Clinical Practices" and the Belgian Law of 7 May 2004 related to experiments on humans.

*Surname of the investigator:* ………………………………………………

*First name of the investigator:* ………………………………………………

*Date:* ………………………………………………

*Signature of the investigator:* ………………………………………………

*Surname of the investigator's representative:* ………………………………………………

*First name of the investigator's representative:* ………………………………………………

*Date:* ………………………………………………

*Signature of the investigator's representative:* ………………………………………………

Titel van de studie: **Het ouderperspectief bij besluitvorming rondom foetale chirurgie**

**III Supplementary information**

**The protection and the rights of the participant in a clinical study :**

***Ethics Committee***

This study has been reviewed by an independent Ethics Committee, namely the Ethics Committee of UZ/KU Leuven, which has issued a favourable opinion. It is the task of the Ethics Committees to protect people who take part in a clinical trial. They make sure that your rights as a patient and as a participant in a clinical study are respected; that based on current knowledge, the balance between risks and benefits remains favourable to the participants and that the study is scientifically relevant and ethical.

You should not under any circumstances take the favourable opinion of the Ethics Committee as an incentive to take part in this study.

***Voluntary participation***

Before signing, do not hesitate to ask any questions you feel are appropriate. Take the time to discuss matters with a trusted person if you so wish.

Your participation in the study is voluntary and must remain free of any coercion: this means that you have the right not to take part in the study or to withdraw without giving a reason, even if you previously agreed to take part. Your decision will not affect your relationship with the investigator or the quality of your future therapeutic care.

However, it is advisable for your safety to inform the investigator if you have decided to stop taking part in the study.

If you agree to take part, you will be asked to sign the informed consent form. The investigator will also sign this form to confirm that he/she has provided you with the necessary information about the study. You will receive a copy of this form.

***Costs associated with your participation***

If you decide to take part in this study, this will not involve any extra costs for you or your insurer, and you will not be reimbursed for your participation.

***Guarantee of confidentiality***

Your participation in the study means that you agree to the investigator collecting data about you and to the study sponsor using these data for research purposes and in connection with scientific and medical publications.

Your data will be processed in accordance with the European General Data Protection Regulation (GDPR) and with the Belgian legislation on the protection of natural persons with regard to the processing of personal data.

You are entitled to ask the investigator what data are being collected about you and what its use is in connection with the study. This data concerns your current clinical situation but also some of your background, the results of examinations carried out and the results of examinations required by the protocol. You have the right to inspect this data and correct it if it is incorrect[1].

The investigator has a duty of confidentiality regarding the data collected.

1 These rights are guaranteed by the Law of 8 December 1992 on the protection of privacy in relation to the processing of personal data and by the Law of 22 August 2002 on patient rights.

This means that he/she undertakes not only to never to reveal your name in the context of a publication or conference but also that he/she will encode your data (i.e. your identity will be replaced by an ID code in the study) before sending it to the manager of the database of collected data (KU/UZ Leuven, Leuven).

The investigator and his/her team will therefore be the only ones to be able to establish a link between the data transmitted throughout the study and your medical records[2].

The personal data transmitted will not contain any combination of elements that might allow you to be identified[3].

For the study data manager designated by the sponsor, the data transmitted will not allow you to be identified. The latter is responsible for collecting the data gathered by all investigators taking part in the study, processing them and protecting them in accordance with the requirements of the Belgian law on the protection of privacy.

To verify the quality of the study, it is possible that your medical records will be examined by persons subject to professional secrecy and designated by the ethics committee, the sponsor of the study or an independent audit body. In any event, this examination of your medical records may only take place under the responsibility of the investigator and under the supervision of one of the collaborators designated by him/her.

The encoded study data will be able to be sent to Belgian or other regulatory authorities, to the relevant ethics committees, to other doctors and/or to organisations working in collaboration with the sponsor.

Your consent to take part in this study therefore also implies your consent to the use of your encoded medical data for the purposes described in this information form and to their transmission to the aforementioned people and authorities.

The sponsor undertakes only to use the data collected within the context of the study in which you are taking part.

If you withdraw your consent to take part in the study, to guarantee the validity of the research, the data encoded up to the point at which you withdraw will be retained. No new data may be sent to the sponsor.

If you have any questions relating to how your data are being processed, you may contact the investigator. The data protection officer in your hospital can be contacted as well: DPO - UZ Leuven, Herestraat 49, 3000 Leuven, e-mail dpo@uzleuven.be.

Finally, if you have a complaint concerning the processing of your data, you can contact the Belgian supervisory authority who ensures that privacy is respected when personal data are processed.

The Belgian supervisory authority is called:

Data Protection Authority (DPA)

Drukpersstraat 35,

1000 Brussels

Tel. +32 2 274 48 00

e-mail: contact@apd-gba.be

Website: https://www.dataprotectionauthority.be

---

[2] For clinical trials, the law requires this link with your records to be retained for 20 years. In the case of an advanced therapy medicinal product using human biological material, this period will be a minimum of 30 years and a maximum of 50 years in accordance with the Belgian Law of 19 December 2008 on the use of human biological material and the applicable royal decrees.

3 The database containing the results of the study will therefore not contain any combination of elements such as your initials, your gender and your full date of birth (dd/mm/yyyy).

***Insurance***

Any participation in a clinical study involves a risk, however small it is. Even if there is no fault, the sponsor accepts responsibility for damage caused to the participant (or in the event of death, his/her dependants) and directly or indirectly linked to his/her participation in the study. The sponsor has taken out insurance for this responsibility[4].

Van Breda Risks & Benefits NV, Plantin en Moretuslei 297, 2140 Antwerpen, polisnummer 299.053.700.

[4] In accordance with Article 29 of the Belgian Law related to experiments on humans (7 May 2004)
