# Supplementary material for: ‘We did everything we could’– a qualitative study exploring the acceptability of maternal-fetal surgery for spina bifida to parents
Source: Prenat Diagn. Author manuscript; Available in PMC 2022 Sep 7. (PMC7613560; doi:10.1002/pd.5996)
Supplement: Supplementary Information [file EMS152847-supplement-Supplementary_Information.zip › pd5996-sup-0003-suppl-data.docx]

**Interview guide**

The aim of the interview is to assess how women (and their partners) perceive the acceptability of a fetal surgical intervention for MMC and CDH, with regards to the GIFT-surg project. Participants will be asked to share their thoughts, views, feelings and experiences with regards to their decision to participate in fetal surgery.

Due to the nature of the interview, the interview style to be used is called ‘responsive’ interviewing(1) This interview style emphasizes on the importance of building a relationship of trust between interviewer and interviewee. Therefore, it is essential to invest on building on this relationship. This is mainly done by creating a quiet and calm environment, an empathically neutral position of the interviewer and an open approach by using both verbal and non-verbal communication (2). The interview will not be set-up as a question-answer setting, but as a setting in which the respondent feels free to share their story, to get an understanding of the respondent’s perceptions, feelings, thoughts, views and experiences. In other words, the respondent needs to do most of the talking. The interviewer will guide her through the key-themes.

*Stages of the interview*

Before recording the interview, the interviewer will introduce her/himself to the interviewee. This must be seen as a normal introduction, when two (or three people) first meet. These first few minutes are crucial for establishing a good *rapport* (2)(3). Building *rapport* means that the interviewer shows to the respondent that she/ he is sincerely interested in her/ their story (STAGE 1). When both interviewer and respondent(s) are at ease, the interviewer will introduce the research and explain the purpose of the interview. It will be emphasized that participating is voluntary, and arrangements of confidentiality will be set out. There is no wrong or right answer, the interviewer is interested the respondent’s perspective, in their own words. The respondents will be informed that if they do not wish to speak about certain areas, they do not have to (STAGE 2). When both interviewer and respondent(s) are at ease, the interviewer will empathically reflect on the personal situation of the respondent(s) and the past few weeks and days in which they have received the diagnosis and all the different options. First, this is to collect some information on their history and context (the interviewer is informed on major details), second this is invite the respondent to reflect on what happened to them. The interviewer will not record this, but will make notes if necessary. This first phase is also used to collect the *contextual questions*, if not already collected (STAGE 3). The actual interview will start then with an open question: “*I would like to learn more on how you have come to the decision for the treatment of your baby: fetal surgery*”. Respondents will be encouraged to share their thoughts, views, feelings and experiences regarding the decision to participate in fetal surgery. By using probing questions, the interviewer further explores the different levels of this decision. The seven components of the *theoretically framework of acceptability* will be used as *key themes* or *prompts* (affective attitude, burden, perceived effectiveness, ethicality, intervention coherence, opportunity costs, and self-efficacy (Table 1)), but any other related themes emerging from the respondent, to seek breadth and depth of coverage (STAGE 4). Shortly before the end of the interview (5 to 10 minutes), the interviewer will signal the approach of the end of the interview, to encourage the respondents to raise anything important. This can be done by simply asking for final thoughts or comments (STAGE 5). The interview will be finished by thanking the respondent for her/his participation, and emphasize on the value of their participation. This is also the time give contact details for further questions, or support services. This is also the moment to discuss how and when we will contact the respondent for the next interview.

| Thoughts and emotions regarding taking part in the intervention | *Affective attitude* |
| --- | --- |
| The perceived amount of effort that is required to participate in the intervention (to much cognitive effort, too much risks, too expensive, too much time) | *Burden* |
| How is the intervention judged by a person’s individual feelings and values? (morally good or correct) | *Ethicality* |
| What is the participant’s understanding of the intervention? | *Intervention coherence* |
| What benefits, profits or values must be given up to engage in the intervention, from the participant’s point of view | *Opportunity costs* |
| What are chances of the intervention being able to cure or improve the condition being treated as perceived by the participant | *Perceived effectiveness* |
| To which extent the participant feels confident to be able to motivate oneself(ves) to participate and to adapt to the behavioral changes required by the intervention | *Self-efficacy* |

*Table 1 Key themes based on components of theoretical framework of acceptability*

Interview identification code: ………….

Initials: ………….

*Demographic variables*

Age: ………….

Parity: ………….

Education: ………….

Marital status: ………….

Number of living children: ………….

Country of origin: ………….

*Variables on fetus/ neonate*

Fetal diagnosis: ………….

Chosen treatment option: ………….

Gestational age of the fetus/ neonate at interview X: …..wks.

Preference to receive study results yes/no

**References**

1. Rubin, H; Rubin I. Qualitative interviewing. The art of hearing data. Thousand Oaks: Sage; 2005.
2. Evers J. Het kwalitatieve interview: kenmerken, typen en voorbereiding. In: Evers J, editor. Kwalitatief interviewen: kunst en kunde. 2nd ed. Amsterdam: Boomlemma; 2015.
3. Yeo, A; Legard, R; Keegan, J; Ward, K; McNaughton Nicholls, C; Lewis J. In-depth interviews. In: Ritchie,J; Lewis, J; McNaughton Nicholls, C; Ormston R, editor. Qualitative Research Practice. Second. London: Sage; 2014.
